# Livestock-Associated MRSA Carriage in Patients without Direct Contact with Livestock

**DOI:** 10.1371/journal.pone.0100294

**Published:** 2014-06-27

**Authors:** Miranda M. L. van Rijen, Thijs Bosch, Erwin J. M. Verkade, Leo Schouls, Jan A. J. W. Kluytmans, on behalf of the CAM Study Group

**Affiliations:** 1 Laboratory for Microbiology and Infection Control, Amphia Hospital, Breda, the Netherlands; 2 Center for Infectious Disease Control Netherlands, National Institute for Public Health and the Environment, Bilthoven, the Netherlands; 3 Laboratory for Medical Microbiology and Immunology, St. Elisabeth Hospital, Tilburg, the Netherlands; 4 Department of Medical Microbiology and Infection ControlJK, VUmc Medical University, Amsterdam, the Netherlands; University of Mississippi Medical Center, United States of America

## Abstract

**Background:**

Livestock-associated MRSA (MC398) has emerged and is related to an extensive reservoir in pigs and veal calves. Individuals with direct contact with these animals and their family members are known to have high MC398 carriage rates. Until now it was assumed that MC398 does not spread to individuals in the community without pig or veal calf exposure. To test this, we identified the proportion of MC398 in MRSA positive individuals without contact with pigs/veal calves or other known risk factors (MRSA of unknown origin; MUO).

**Methods:**

In 17 participating hospitals, we determined during two years the occurrence of MC398 in individuals without direct contact with livestock and no other known risk factor (n = 271) and tested in a post analysis the hypothesis whether hospitals in pig-dense areas have higher proportions of MC398 of all MUO.

**Results:**

Fifty-six individuals (20.7%) without animal contact carried MC398. In hospitals with high pig-densities in the adherence area, the proportion of MC398 of all MUO was higher than this proportion in hospitals without pigs in the surroundings.

**Conclusions:**

One fifth of the individuals carrying MUO carried MC398. So, MC398 is found in individuals without contact to pigs or veal calves. The way of transmission from the animal reservoir to these individuals is unclear, probably by human-to-human transmission or by exposure to the surroundings of the stables. Further research is needed to investigate the way of transmission.

## Introduction

Since 2003, the so-called livestock-associated MRSA (LA-MRSA) has emerged in animals and humans in areas with intensive animal farming in Europe, North America, and Asia [1]. Human carriage of LA-MRSA is strongly related to direct contact with pigs, veal calves and broilers [2], [3]. The majority of these LA-MRSA strains belong to multilocus sequence type clonal complex 398 (CC398) [4]. After its emergence, the risk factor ‘direct contact with living pigs, veal calves and broilers’ was added to the Dutch national MRSA guideline and an active screening program in hospitals was implemented [5]. By the end of 2011, 39% of all newly identified MRSA strains in humans in the Netherlands belonged to this variant in the Netherlands [6].

Recent surveys showed that MRSA CC398 was 4 to 6-fold less transmissible than other MRSA strains in a hospital-setting [7]–[8]. At present, the human-to-human transmissibility of MRSA CC398 in a community setting is still unclear. Considering the extensive reservoir in animals and people who work with livestock, the occurrence of MRSA CC398 in people who are not directly involved in farming is strikingly low. So far, there are no indications that MRSA CC398 has spread extensively into the general population [9]. A cross-sectional survey in a livestock-dense region found that only 0.2% of adult individuals without livestock contact were positive for MRSA CC398 [10]. On the other hand, there are observations that proximity of farms is a potential risk factor, even in absence of direct contact between humans and animals [11]–[13]. In addition, in a recent exploratory study an association was found between consumption of poultry and MRSA carriage [14]. A spectrum of infections with MRSA CC398 have been documented, ranging from relatively minor or localized infections including abscesses [15]– and various skin and soft tissue infections (SSTI) [18]–[20], urinary tract infections [16], wound infections [16], mastitis [4], and conjunctivitis [21], as well as more serious or invasive infections, including bacteremia [21]–[24], endocarditis [24], [25], pneumonia (including necrotizing pneumonia, osteomyelitis, pyomyositis, and postoperative infections [26]. Despite the diverse array of infection types reported, it has been suggested that MRSA CC398 is less virulent than other human MRSA strains [27].

Apart from LA-MRSA and hospital-associated (HA)MRSA, MRSA rates also are rapidly increasing in community dwelling individuals without known healthcare- or livestock-associated risk factors. This third entity has been referred to as community-acquired (CA) MRSA [28] or MUO [29]. In this study, the proportion of CC398 in MUO isolates was determined. We hypothesized that people living in an area in which CC398 is common have more risk of MRSA CC398 carriage than persons living in an area in which CC398 is rare.

## Methods

### Ethics Statement

Ethical approval for the study was obtained by the medical ethics committee of the St. Elisabeth Hospital in Tilburg (NL 19489.008.07, protocol 0749, March 9^th^, 2009). Patient information was anonymized and de-identified prior to analysis.

### MRSA source identification

To identify MRSA sources in the Netherlands, Infection Control Practitioners (ICP) from seventeen hospitals (three academic, seven teaching and seven general hospitals) throughout the Netherlands were asked to complete a questionnaire on a website for all consecutive patients that were found to be MRSA positive (both infection and carriage) for the first time in the microbiological laboratory of the hospital from January 2009 until December 2010. Samples were taken during a visit to the outpatient's clinic or during a stay on a ward in the hospital. Patients who had already been found MRSA-positive in the past were not included. The questionnaire on the website contained data about patient type (in- or outpatient), demographics, positive body sites, molecular typing results and probable source of MRSA. The MRSA source was identified based on the patient's history combined with molecular typing results and then classified in risk groups described in the national infection prevention guidelines [5]. When neither of these risk groups was applicable, the MRSA was classified as ‘MRSA of unknown origin (MUO)’.

### Genotyping of MRSA isolates

All MRSA isolates were genotyped by multiple-locus variable number of tandem repeat analysis (MLVA) by the Dutch National Reference Center (RIVM, Bilthoven, the Netherlands) [30]. MLVA is known for its higher discriminatory power for LA-MRSA strains as compared to either multilocus sequence typing (MLST) or pulsed-field gel electrophoresis (PFGE) [30]. The MLVA profiles were clustered using a categorical clustering coefficient (unweighted-pair group method using arithmetic averages, UPGMA) and a minimum spanning tree was constructed to display the relationships between the various MLVA complexes (MC) and MRSA sources. For this study, we incorporated phiSa3 into the MLVA scheme. Furthermore, tetM was determined by use of DNA microarray (Identibac *S. aureus* Genotyping, Alere).

### Data analysis

The percentage of MC398 in the group with individuals not reporting contact with pigs or veal calves was determined. We hypothesized that individuals without direct contact with pigs/veal calves living in a pig-dense area have more chance to become colonized with MC398 MUO than individuals living in areas without many pigs. Hospitals were divided into two categories: 1) Hospital with an adherence area with a high pig-density; 2) Hospital with an adherence area with a low pig-density. Municipality level data of the number of pigs were downloaded from the website of the Central Institute for Statistics (CBS) [31]. To test our hypothesis, the numbers of MC398 MUO positive individuals in these two categories were compared in a Chi-square test in a post analysis. To avoid bias by possible different screening policies of the 17 different hospitals, only MRSA infections were included in this analysis. In this way, unexpected findings in contract tracings were excluded.

## Results

During 2009–2010, 1020 patients (368 inpatients and 652 outpatients) were found to be MRSA-positive in the seventeen participating hospitals. From 299 (29.3%) patients, MRSA-positive samples were obtained from body sites other than nose, throat, and perineum, mainly urine, sputum and wounds. Eight patients suffered from a bacteremia with MRSA (0.8%). In 39 patients (3.8%), MRSA was found in the perineum sample only, while other tested sites were found to be negative for MRSA.

**Figure 1:**
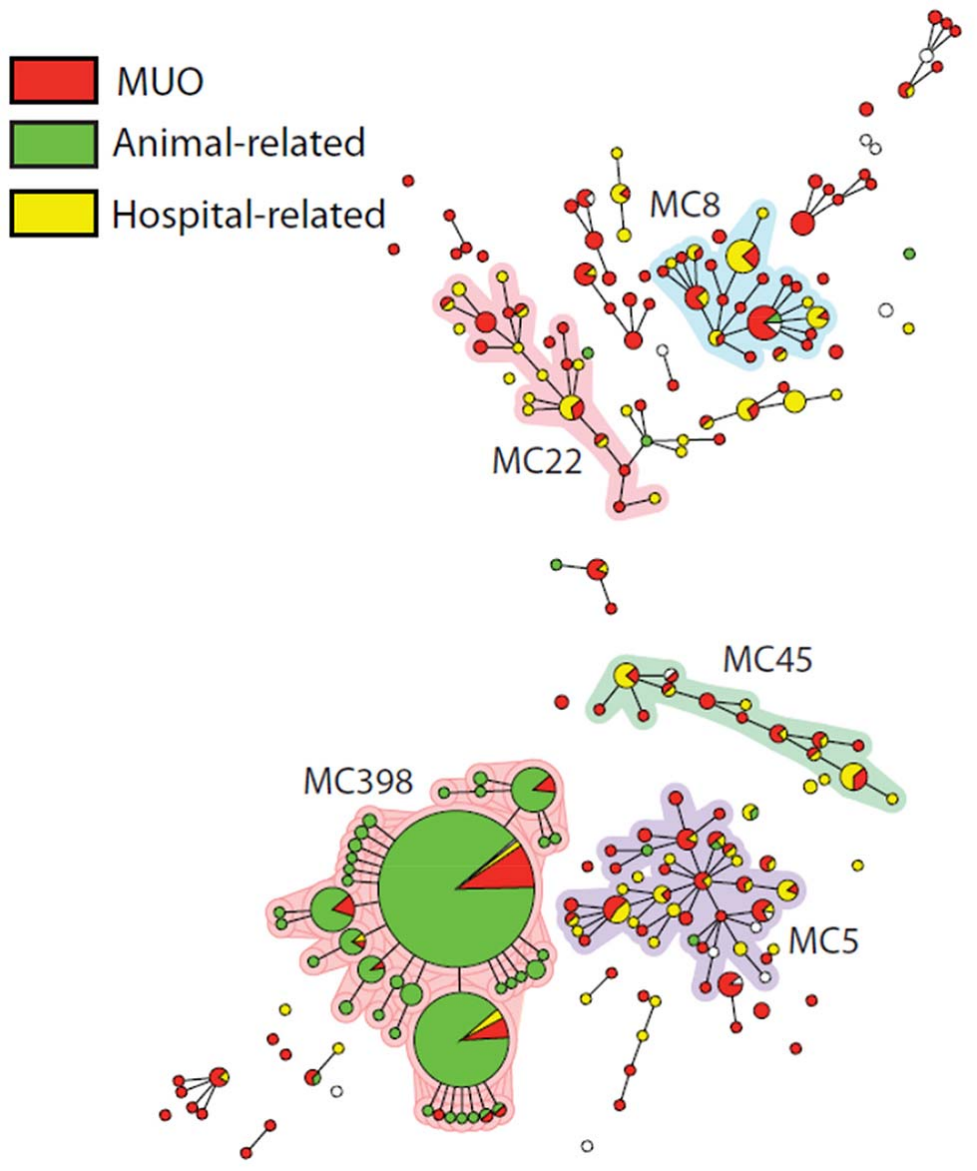
Genotypic relatedness of 1020 MRSA isolates represented as a minimum spanning tree based on MLVA types. Clustering of MLVA profiles was obtained using a categorical coefficient to create a minimum spanning tree in which the MLVA types are displayed as circles. The size of each circle indicates the number of isolates with this particular type. MLVA complexes (MC) are indicated in characters e.g. MC398 denotes MLVA complex 398.

**Figure 2:**
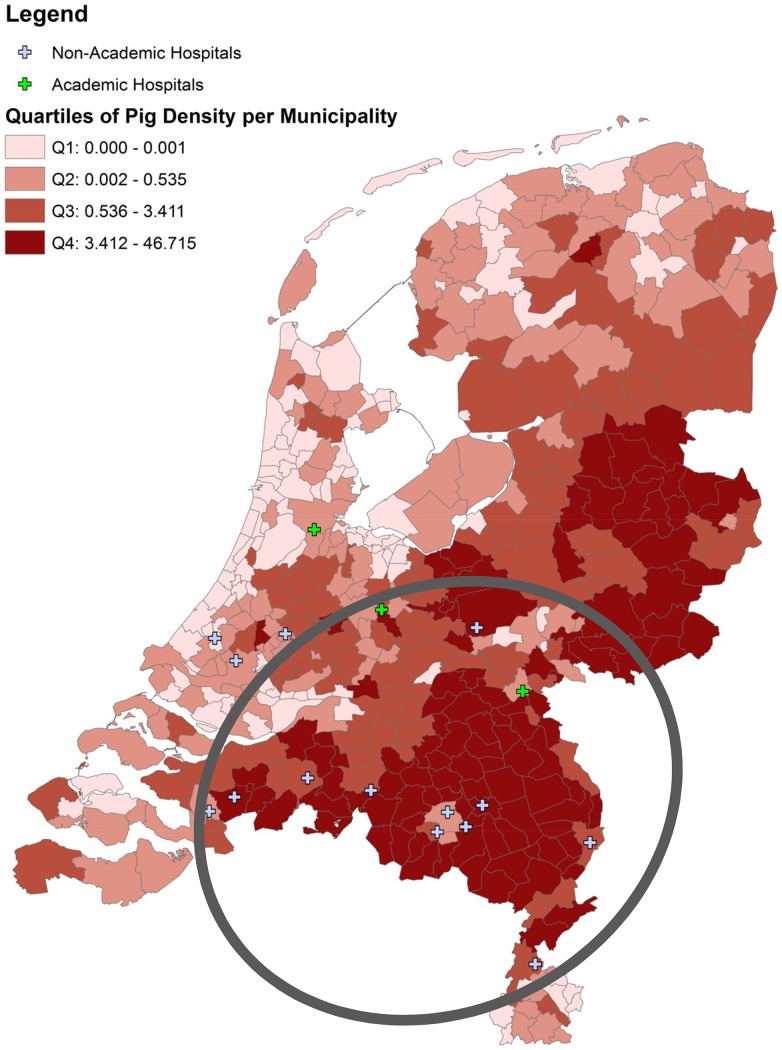
Pig-densities in the Netherlands. Hospitals with high pig-densities in the adherence areas are presented within the circle.

MRSA source analysis is depicted in Table 1. MLVA typing of the strains showed that 649/1020 (63.6%) strains were MC398. Two-hundred and seventy one (26.6%) of all newly identified carriers were of unknown origin, and 56 (20.7%) of them were MC398. These 56 MC398 isolates were tetM positive and lacked the prophage Sa3 (phiSa3). The mean risk to find a MC398 MUO in a participating hospital was estimated at 1 per 8 months (1 per 12 months for infections only). Thirty-five of the 56 (62.5%) individuals suffered from an infection. Figure 1 shows MUO, hospital- and animal-related MRSA and their MLVA complexes. MC398 MUO and MC398 of patients with animal contact cluster together. To test our hypothesis that individuals without animal contact have more chance to carry MC398 MRSA in pig-dense areas than in areas without many pigs, a Chi-square test was performed for hospitals with an adherence area with many pig farms compared to hospitals in an area without many pigs. Data of all participating hospitals is shown in Table 2. Pig-densities in the Netherlands are shown in Figure 2. We found an indication that, in hospitals with high pig-densities in the adherence area, the proportion of MC398 infection of all MUO infection is higher than in hospitals without pigs in the surroundings (32/148 vs. 3/59; RR 4.25 95% CI 1.35–17.21, *P* = 0.004).

**Table 1:** MRSA sources in patients in 17 Dutch hospitals, 2009–2010.

Source	Total		MC398	
	N	% of total	n	% within source
Pigs/veal calves	603	59.1	587	97.3
Foreign hospital	75	7.4	3	4.0
Nosocomial transmission	44	4.3	3	6.8
Transmission in nursing home	5	0.5	0	0.0
Adoption children	18	1.8	0	0.0
Dialysis patients from foreign countries	2	0.2	0	0.0
Unknown origin (MUO)	271	26.6	56	20.7
No data	2	0.2		
**Total**	**1020**	**100**	**649**	**63.6**

**Table 2:** Unknown risk factor (MUO) and proportion of MC398 within this group, shown per hospital.

Hospital	Hospital type	Newly identified MRSA (total)	Pig-density in adherence area	Unknown risk factor (MUO)		MC398 MUO	
				n_total_ (n_infection_)	%	n_total_ (n_infection_)	%
1	teaching	100	High	39 (32)	39.0	7 (5)	17.9
2	teaching	53	High	10 (7)	18.9	2 (2)	20.0
3	general	95	High	10 (7)	10.5	5 (5)	50.0
4	general	137	High	24 (18)	17.5	9 (6)	37.5
5	general	26	Low	17 (11)	65.4	2 (1)	11.8
6	general	19	High	4 (3)	21.1	0	0
7	teaching	54	High	6 (6)	11.1	1 (1)	16.7
8	general	30	High	19 (16)	63.3	1 (1)	5.3
9	teaching	18	Low	9 (9)	50.0	1 (0)	11.1
11	general	40	High	5 (5)	12.5	1 (1)	20.0
12	teaching	84	High	15 (9)	17.9	4 (0)	26.7
13	teaching	25	Low	18 (15)	72.0	1 (1)	5.6
14	academic	60	High	23 (16)	38.3	12 (9)	52.2
15	academic	48	High	23 (14)	47.9	6 (1)	26.1
16	academic	52	Low	30 (24)	57.7	1 (1)	3.3
17	general	26	High	9 (9)	34.6	2 (1)	22.2
18	teaching	151	High	10 (6)	6.6	1 (0)	10.0
**Total**		**1018** a		**271 (207)**	26.6	**56 (35)**	20.7

a **in two individuals there were no data about the source.**

**Hospital 10 intended to participate, but completed no electronic forms.**

## Discussion

The majority (n = 603, 59.1%) of newly identified MRSA-positive patients in 17 hospitals in 2009 and 2010 was related to exposure to livestock. A substantial proportion could not be classified to an established risk group (n = 271, 26.6%) and are therefore assumed to have acquired their MRSA in the community. One fifth (20.7%) of these MRSA strains belonged to MC398. The presence of the tetM resistance gene and the absence of the phiSa3 suggest that these isolates were animal-associated [22], [32]. We found an indication that, in hospitals with high pig-density in the surroundings, the proportion of MC398 infection of all MUO infection was higher than in hospitals with a low pig-density in the surroundings. This indicates that LA-MRSA may be spreading through other sources than direct exposure to livestock. Until now it was assumed that LA-MRSA is able to spread to the pig/veal calf farmers and others who are in close contact with the animals, but is less able to spread from the farmer to household members who do not enter the stables, and is almost unable to spread to persons in the community without pig or veal calf exposure. Thus, it is assumed that constant pressure of LA-MRSA from animals with MRSA must be present to maintain the LA-MRSA colonization in humans. However, several recent studies have shown that persistent colonization with MC398 is possible [33]–[35]. Moreover, pig-, dairy cow, and veal calf densities per municipality were also found to be independent risk factors for carriage of MRSA MC398 in two recently published case-control studies [11], [14]. Although it cannot be excluded that human-to-human transmission occurs in areas with a high MRSA MC398 pressure, environmental contamination with MRSA MC398 may play a role as well. MRSA MC398 has been shown to be present in air and soil samples collected downwind of pig and swine barns [13]. Other transmission routes can play a role as well. For example, regular consumption of poultry was recently found to be associated with CA-MRSA transmission in an exploratory hospital-based case-control study [14]. De Boer et al. demonstrated that a substantial part of the meat products obtained from retail stores in the Netherlands were colonized with MRSA, including both MC398 and non-MC398 strain types [36]. However, meat consumption cannot explain the increased prevalence in people who live in pig-dense areas. We expect the risk, associated with meat consumption, to be the same for all areas over the country. Unless, locals consume more meat from their own area.

### Limitations

We performed a post hoc analysis to study whether the proportion of MC398 MUO infection is higher in hospitals in pig-dense areas than in areas with a low pig-density. Our study was originally not designed for this purpose. Therefore, we have to be careful with the conclusions. An analysis in which pig-density was determined based on postal code of the individuals would have been more reliable. These data were not available because of privacy issues. Also, the stratification of hospitals in ‘pig-dense’ and ‘pig-arm’ areas is arbitrary. Based on the CBS data, we classified the hospitals that are known to be situated in the most urbanized parts of the country as ‘pig-arm’. This resulted in four hospitals in pig-arm areas and 13 hospitals in pig-dense areas. So, more hospitals in pig-dense areas were included in the analysis. Furthermore, there may be detection bias due to differences in screening policies between hospitals. It is possible that physicians in some hospitals take more clinical samples than physicians in other hospitals. This may lead to an underestimation in the number of MUO findings. Also, classification bias may occur depending on the reliability of the history of risk factors. However, all participating hospitals screened the MRSA risk groups described in the national MRSA guideline [5]. After coincidental MRSA findings, patients were asked for these risk factors also.

In conclusion, this study shows that the majority of newly identified MRSA patients in these 17 hospitals were acquired by direct contact with pigs/veal calves. The second largest group is the group of unknown origin. One fifth of these MUO are MC398. We found a significant association between individuals living in pig-dense areas and the likelihood of MC398 MUO carriage. MC398 MUO infections were rarely detected, i.e. 1 per 12 months for every participating hospital, so, currently, this MC398 MUO seems not to cause many problems. Because of the absence of known risk factors and probable risk for transmission in the healthcare settings, it is worthwhile to monitor the number of MUO in general, and of MC398 separately, in the coming years.
